# A Rare Clinical Encounter: Loculated Pleural Effusion in a Young Male With a History of Resected Cutaneous Melanoma

**DOI:** 10.7759/cureus.83911

**Published:** 2025-05-11

**Authors:** Ali T Alhashem, Mohammed S Almulaify, Hussain S Aldahmen

**Affiliations:** 1 Internal Medicine, Dammam Medical Complex, Dammam, SAU; 2 Internal Medicine, Almana Hospital, Dammam, SAU; 3 Pulmonary Medicine, Dammam Medical Complex, Dammam, SAU

**Keywords:** breast melanoma in male, malignant melanoma metastasis, pleural effusion, pleural melanoma, primary cutaneous melanoma

## Abstract

Melanoma is known as one of the most aggressive types of malignant skin carcinoma. The development of melanoma is most commonly associated with excessive sun exposure, a family history of the disease, and genetic predisposition. Melanoma can metastasize to various anatomical sites; the lungs are a frequent site of metastatic involvement, and when pulmonary metastasis develops, respiratory failure becomes the leading cause of mortality. The predominant pathway of metastatic spread in primary cutaneous melanoma is through the regional lymph nodes. We report a case of a young male with a history of resected cutaneous melanoma one year prior to presentation, who developed gradually worsening shortness of breath and pleuritic chest pain over two weeks. An initial chest X-ray revealed a unilateral pleural effusion, and subsequent pleural tapping confirmed the presence of exudative fluid. Further evaluation with a chest CT scan demonstrated a left-sided loculated pleural effusion and a lobulated nodule with spiculated margins in the upper outer quadrant of the left breast, prompting additional evaluation via mammography and tissue biopsy. ‎Mammography revealed a dense, lobulated mass in the upper left breast, measuring 2 × 1.5 cm and located approximately 2.7 cm from the nipple. An ultrasound-guided biopsy of the lesion was performed. Histopathological analysis showed infiltrating large atypical cells and numerous pigmented cells. Immunostaining revealed positive staining for mesothelial cells (HBME-1) and a high proliferative index (Ki-67). Following pleural aspiration and gradual drainage of pleural fluid over several days, the patient exhibited marked clinical improvement. The case was referred to a tertiary care facility for further staging and comprehensive management. This case highlights the importance of maintaining a high index of suspicion for pleural metastatic melanoma in patients with a history of resected cutaneous melanoma.

## Introduction

Melanoma, a highly perilous malignancy primarily affecting the skin, can originate from various anatomical locations, including the skin, ocular regions, and mucosal surfaces. Recognized as one of the most lethal forms of skin cancer, melanoma significantly contributes to cancer-related mortality, largely due to its pronounced propensity for metastasis [1]. Invasive melanomas account for approximately 1% of all skin cancer cases but are responsible for more than 75% of skin cancer-related fatalities [2]. Numerous studies have demonstrated that the number of melanocytic nevi, a family history of melanoma, and genetic susceptibility are primary risk factors for melanoma development. However, individuals with frequent and prolonged exposure to high levels of sunlight are at the highest risk [3].

Male breast neoplasm is an exceedingly rare malignancy compared to its incidence in females, with a reported ratio of one case in men for every 100 cases in women, constituting approximately 1% of all breast cancer diagnoses. Similarly, cutaneous malignant melanoma of the breast is a relatively uncommon neoplasm, comprising less than 5% of all malignant melanoma cases [4,5]. The clinical presentation of early-stage breast malignant melanoma typically includes the formation of a breast lump. In its initial phase, the condition is characterized by a painless mass with indistinct margins, firm consistency, limited mobility, and progressive tumour enlargement [6]. Melanoma can metastasize to various anatomical sites, including the liver, skeletal system, brain, and lungs. The lungs are a common site of metastatic involvement, and when pulmonary metastasis occurs, respiratory failure becomes the leading cause of mortality. Metastatic melanoma is estimated to affect 0.9 out of every 100,000 individuals [7].

Melanoma cells exhibit distinct patterns of metastatic dissemination. A comprehensive investigation conducted by the German Central Malignant Melanoma Registry on patients diagnosed with primary cutaneous melanoma and subsequent metastasis revealed that the predominant pathway of metastatic spread is via regional lymph nodes, accounting for 50% of cases. Additionally, 22% of patients developed satellite or in-transit metastases, while 28% exhibited distant metastases, including distant cutaneous metastatic sites [8].

The treatment of metastatic melanoma continues to pose considerable challenges. In contrast, the management of early-stage melanoma is predominantly surgical, involving excision of the primary tumor with a 1-2 cm margin, supplemented by radical lymphadenectomy when metastasis is detected in the sentinel lymph nodes. Systemic therapy remains the primary treatment modality for metastatic melanoma [9].

In this case study, we describe a young male patient with a history of a solitary cutaneous postauricular melanoma, which was surgically resected one year prior to his current presentation. The patient sought medical attention at the ED, reporting a one-week history of pleuritic chest pain and exertional dyspnea. A CT scan revealed a sizable left-sided loculated pleural effusion and a lobulated nodule in the left breast. Subsequently, a biopsy of the breast nodule was performed, and histopathological analysis confirmed the presence of invasive melanoma.

## Case presentation

We document the case of a 45-year-old male patient with a prior medical history of resected solitary cutaneous postauricular melanoma, surgically treated one year before his current presentation. At that time, he was not receiving any ongoing treatment. However, he presented to the emergency department with a two-week history of exertional dyspnea, which had an insidious onset and was accompanied by pleuritic chest pain and a non-productive cough.

Upon clinical evaluation, the patient was conscious and alert. Vital signs were recorded as follows: body temperature of 37.4°C, pulse rate of 90 beats per minute, oxygen saturation of 85% on room air (improving to 95% with the administration of 2 liters of oxygen via nasal cannula), and blood pressure of 145/80 mmHg. Examination of the neck revealed a scar on the left postauricular region from the prior melanoma resection; no jugular vein distension or additional lymphadenopathy was noted. Thoracic assessment showed no visible deformities; however, auscultation revealed reduced air entry on the left side, and percussion elicited stony dullness across the entire left lung field. Abdominal examination demonstrated a soft, non-tender abdomen without organomegaly. No edema was observed in the lower extremities bilaterally.

**Figure 1:**
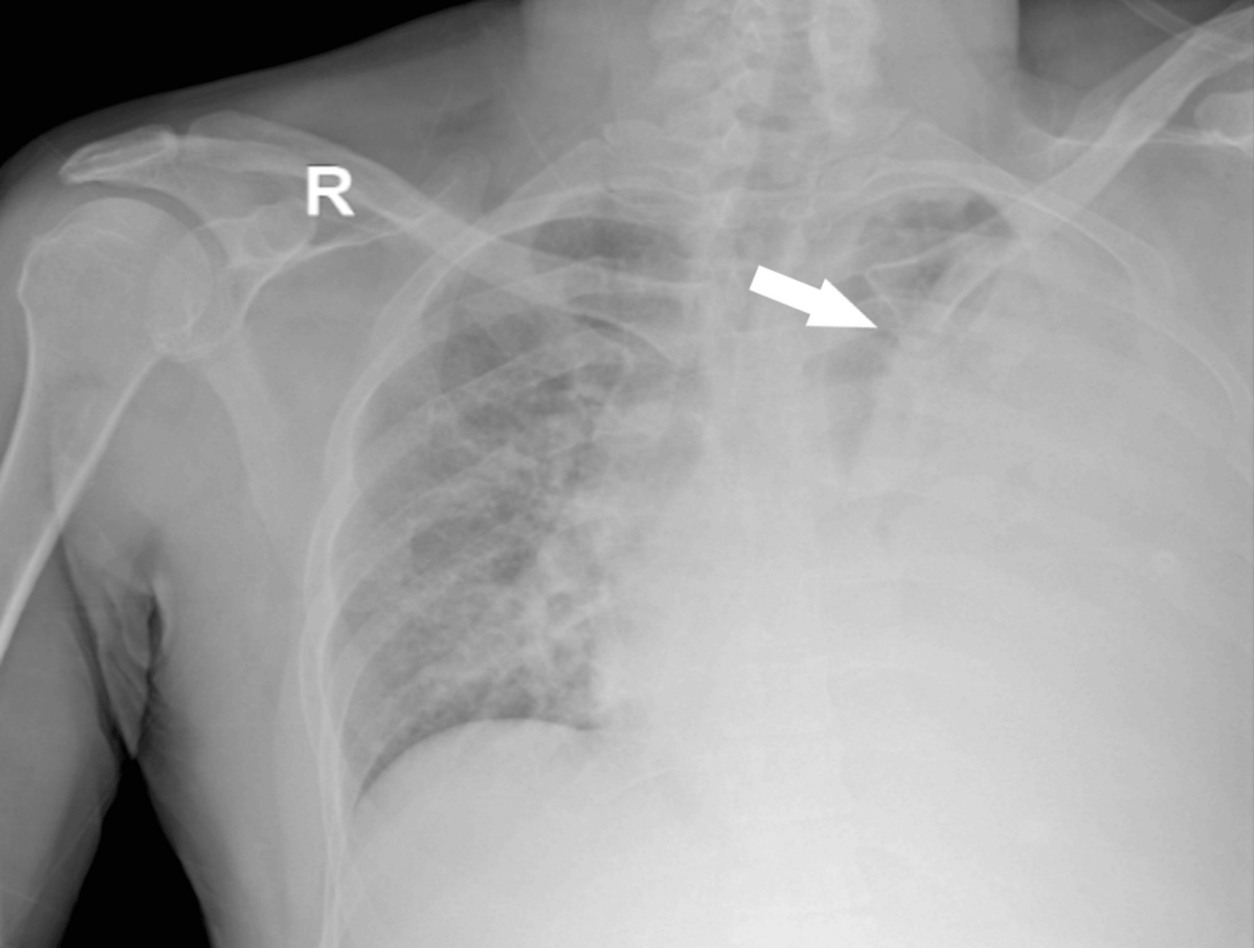
Initial chest X-ray. Chest X-ray showing diffuse opacity throughout the left lung, with sparing of a small apical segment.

Initial diagnostic workup included venous blood gas analysis, which demonstrated a pH of 7.37, partial pressure of carbon dioxide (pCO₂) of 43.3 mmHg, partial pressure of oxygen (pO₂) of 39.1 mmHg, lactate concentration of 0.8 mmol/L, and bicarbonate (HCO₃) level of 24 mmol/L (Table 1). Hematological analysis via complete blood count revealed a hemoglobin level of 11.3 g/dL, WBC count of 19.4 × 10³/µL, platelet count of 430 × 10³/µL, neutrophil count of 15.3 × 10³/µL, and lymphocyte count of 1.37 × 10³/µL. Initial chest X-ray imaging demonstrated diffuse opacity throughout the left lung, sparing a minor apical segment (Figure 1). A provisional diagnosis of pleural effusion, likely of infectious origin, was made, pending confirmation through pleural aspiration and subsequent fluid analysis, including culture with Gram staining, biochemical profiling, Acid-Fast Bacilli (AFB) evaluation, and GeneXpert assay. Empirical treatment with intravenous piperacillin-tazobactam (4.5 g every six hours) was initiated, along with venous thromboembolism prophylaxis and analgesia as needed.

**Table 1:** Venous blood gas analysis.

Parameter	Patient Value	Normal Range
pH	7.37	7.35-7.45
pCO₂ (Partial pressure of CO₂)	43.8 mmHg	35-45 mmHg
pO₂ (Partial pressure of O₂)	39.1 mmHg	80-100 mmHg
Bicarbonate (HCO₃⁻)	24.0 mmol/L	22-26 mmol/L
Base Excess (cBase [Ecf])	+0.4 mmol/L	–2 to +2 mmol/L
Lactate (cLac)	0.8 mmol/L	0.5-2.2 mmol/L
Hemoglobin (ctHb)	10.7 g/dL	13.5-17.5 g/dL (male)
Oxygen Saturation (sO₂)	64.90%	95-100%
Fraction of Oxygenated Hb (FO₂Hb)	63.80%	>94%
Carboxyhemoglobin (FCOHb)	0.90%	<2% (non-smoker)
Potassium (cK⁺)	4.7 mmol/L	3.5-5.0 mmol/L
Sodium (cNa⁺)	137 mmol/L	135-145 mmol/L
Chloride (cCl⁻)	105 mmol/L	98-106 mmol/L
Temperature	37.0°C	36.5-37.5°C

Upon admission, both therapeutic and diagnostic pleural tapping procedures were performed. Pleural fluid analysis yielded the following results: pH of 8, lactate dehydrogenase (LDH) of 750 U/L (compared to serum LDH of 230 U/L), total protein concentration of 54 g/dL (compared to serum protein of 35 g/dL), and glucose concentration of 0.02 mmol/L. The pleural fluid appeared yellow and turbid, with a WBC count of 14,000 cells/mm³, RBC count of 3,000 cells/mm³, neutrophil proportion of 94%, and mononuclear cell proportion of 9% (Table 2). Microbiological assessments, including pleural fluid culture and Gram staining, were negative, as were tests for AFB and GeneXpert analysis.

**Table 2:** Pleural tapping analysis.

Parameter	Value	Normal Range
pH	8	7.60-7.64 (pleural fluid)
Lactate Dehydrogenase (LDH)	750 U/L	<200 U/L (pleural fluid)
Serum LDH	230 U/L	140-280 U/L (serum)
Total Protein	5.4 g/dL	2.0-3.0 g/dL (pleural fluid)
Serum Protein	3.5 g/dL	6.0-8.0 g/dL (serum)
Glucose Concentration	0.02 mmol/L	2.5-4.5 mmol/L (pleural fluid)
Appearance of Pleural Fluid	Yellow and turbid	Clear and pale yellow
WBC Count	14,000 cells/mm³	0-300 cells/mm³
RBC Count	3,000 cells/mm³	0-100 cells/mm³
Neutrophil Proportion	94%	<50% (typically lower in pleural fluid)
Mononuclear Cell Proportion	9%	50-70% (usually more common)

**Figure 2:**
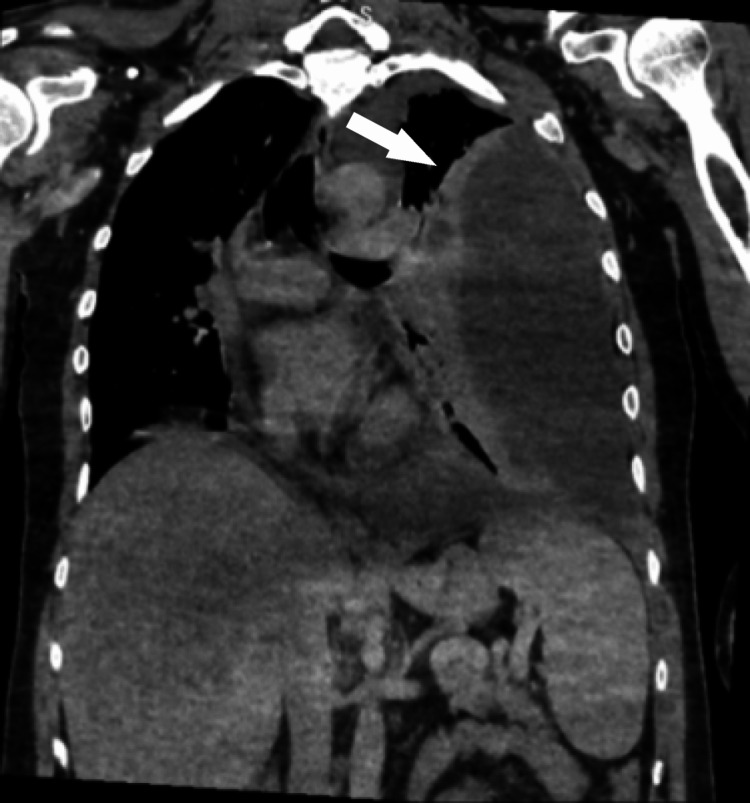
Coronal CT scan of the chest. Coronal CT image of the chest reveals a significant loculated left pleural effusion with mild, smooth pleural thickening and enhancement.

**Figure 3:**
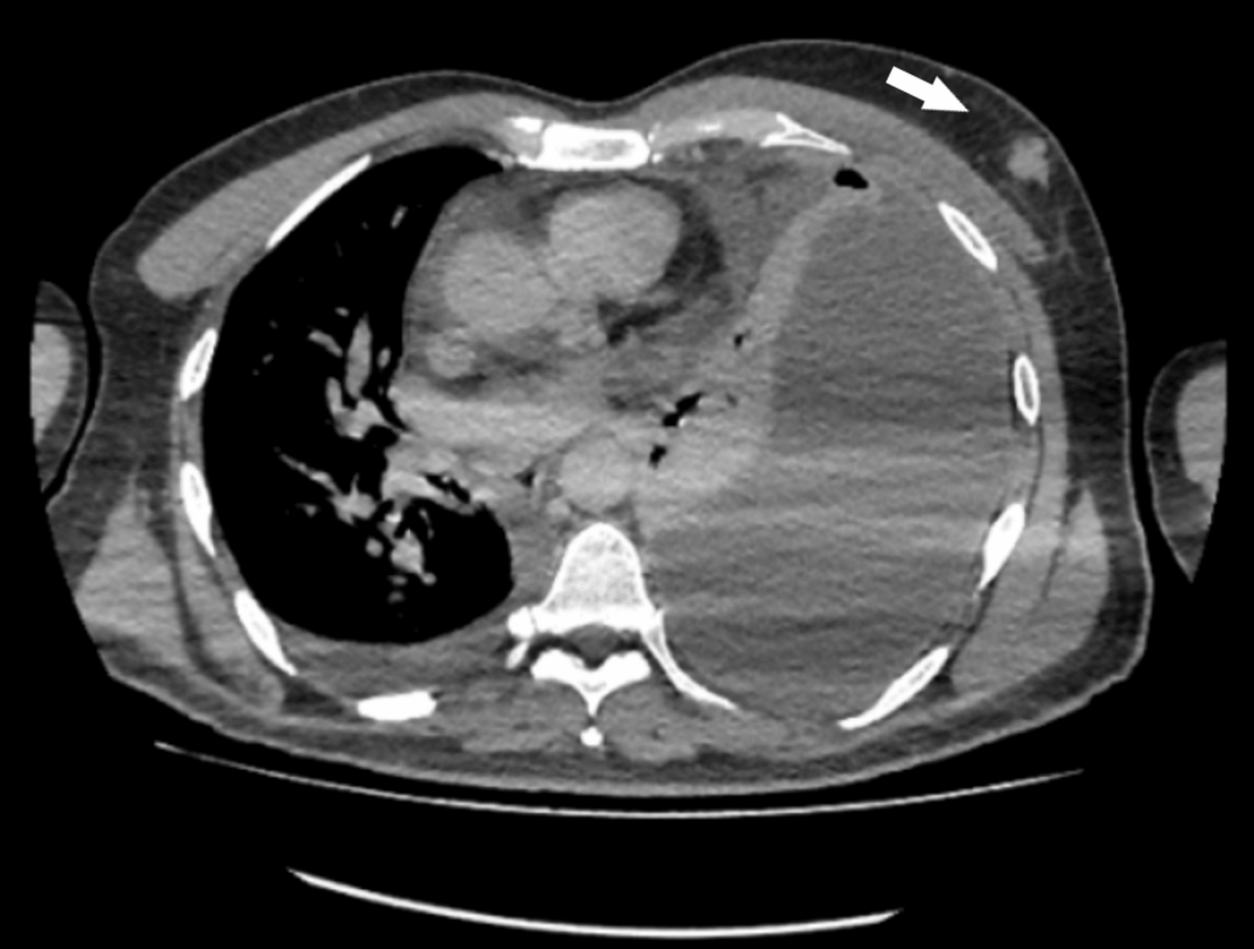
Axial CT scan of the chest. Axial CT image of the chest demonstrates a lobulated nodule with spiculated margins in the upper outer quadrant of the left breast, measuring 1.7 × 1.4 cm, adjacent to a large left-sided pleural effusion.

**Figure 4:**
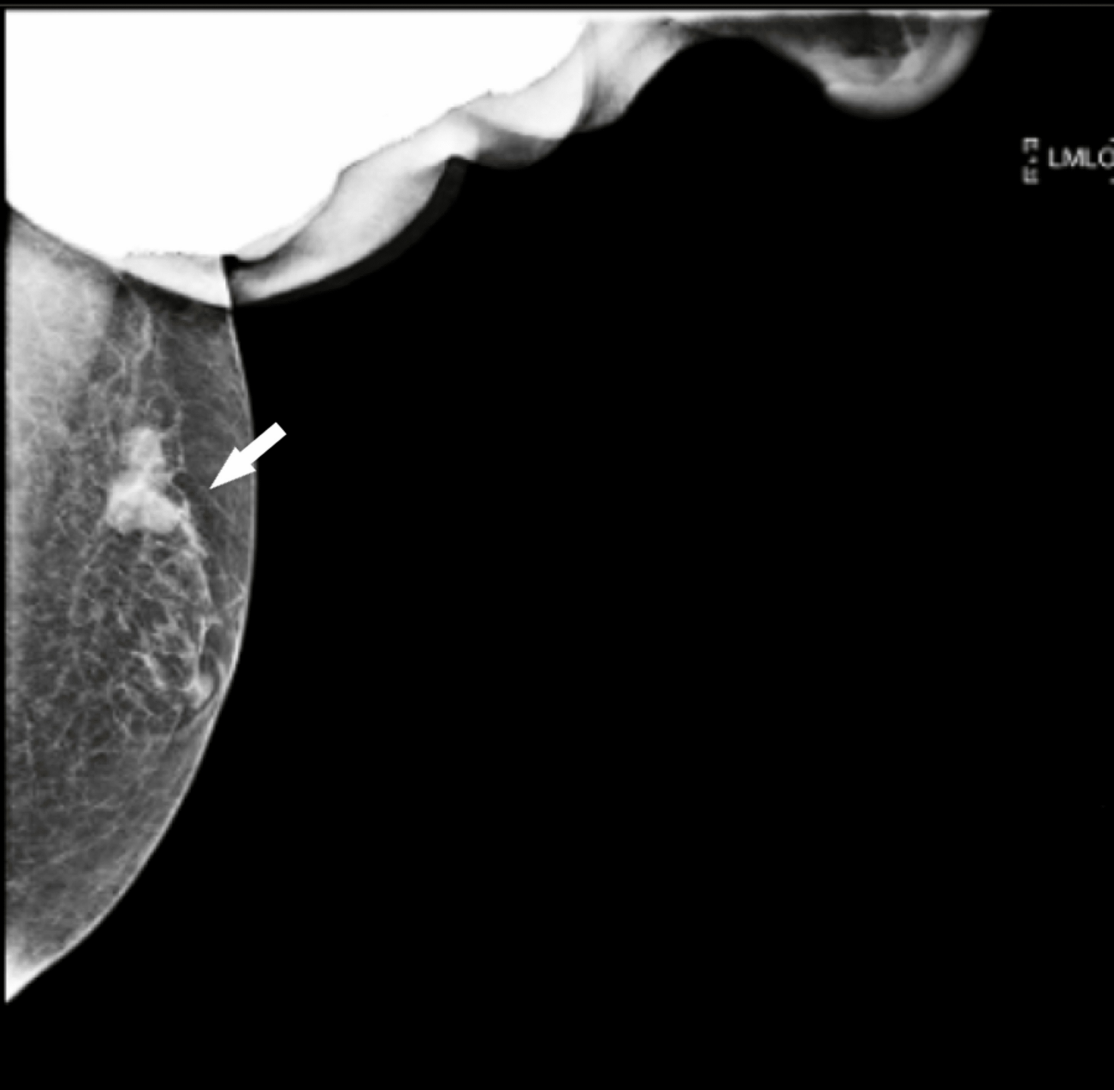
Mammogram of the left breast. The mammogram shows a dense, lobulated mass in the upper left breast measuring 2 × 1.5 cm, located approximately 2.7 cm from the nipple. The left nipple and retroareolar complex appear unremarkable.

Following pleural tapping, a CT scan of the chest was performed, revealing a substantial loculated left pleural effusion with subtle, smooth thickening and enhancement of the pleura. Additionally, multiple indeterminate pulmonary nodules, passive atelectasis, and complete collapse of the left lung were noted. A lobulated nodule with spiculated margins was observed in the upper outer quadrant of the left breast, measuring 1.7 × 1.4 cm, along with involvement of the left axillary lymph nodes (maximum size 0.3 cm), warranting further evaluation via mammography and tissue biopsy (Figures 2-3). Mammography revealed a dense, lobulated mass in the upper left breast, measuring 2 × 1.5 cm and located approximately 2.7 cm from the nipple. The left nipple and retroareolar complex appeared unremarkable. The findings were assigned a Breast Imaging Reporting and Data System (BI-RADS) score of 4C, indicating the need for tissue biopsy to evaluate the suspicious lesion (Figure 4).

**Figure 5:**
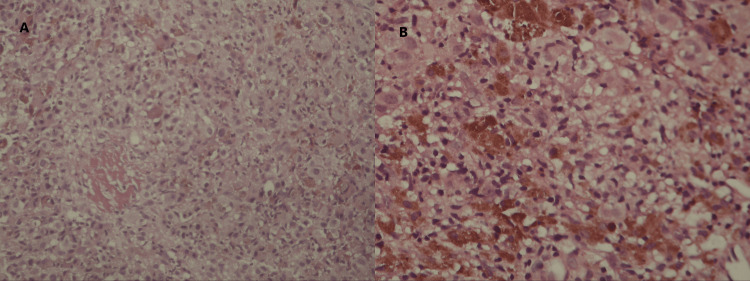
H&E-stained slides. Slide (A) demonstrates infiltration by large atypical cells. Slide (B) shows numerous pigmented cells.

**Figure 6:**
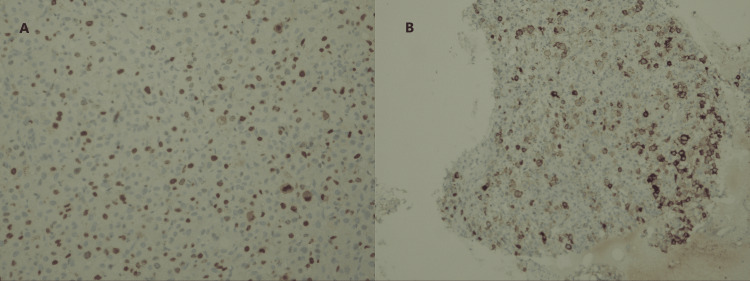
Immunohistochemical staining. Slide (A) demonstrates a high proliferative index (Ki-67). Slide (B) shows positive staining for mesothelial cells (HBME-1).

An ultrasound-guided biopsy of the left breast lesion was performed, and the obtained sample was submitted for histopathological evaluation. H&E-stained slides revealed infiltrating large atypical cells and numerous pigmented cells (Figure 5). Subsequent immunostaining demonstrated positive staining for mesothelial cells (HBME-1) and a high proliferative index (Ki-67) (Figure 6), indicating increased cellular proliferation. Based on these findings, a diagnosis of invasive melanoma was confirmed.

**Figure 7:**
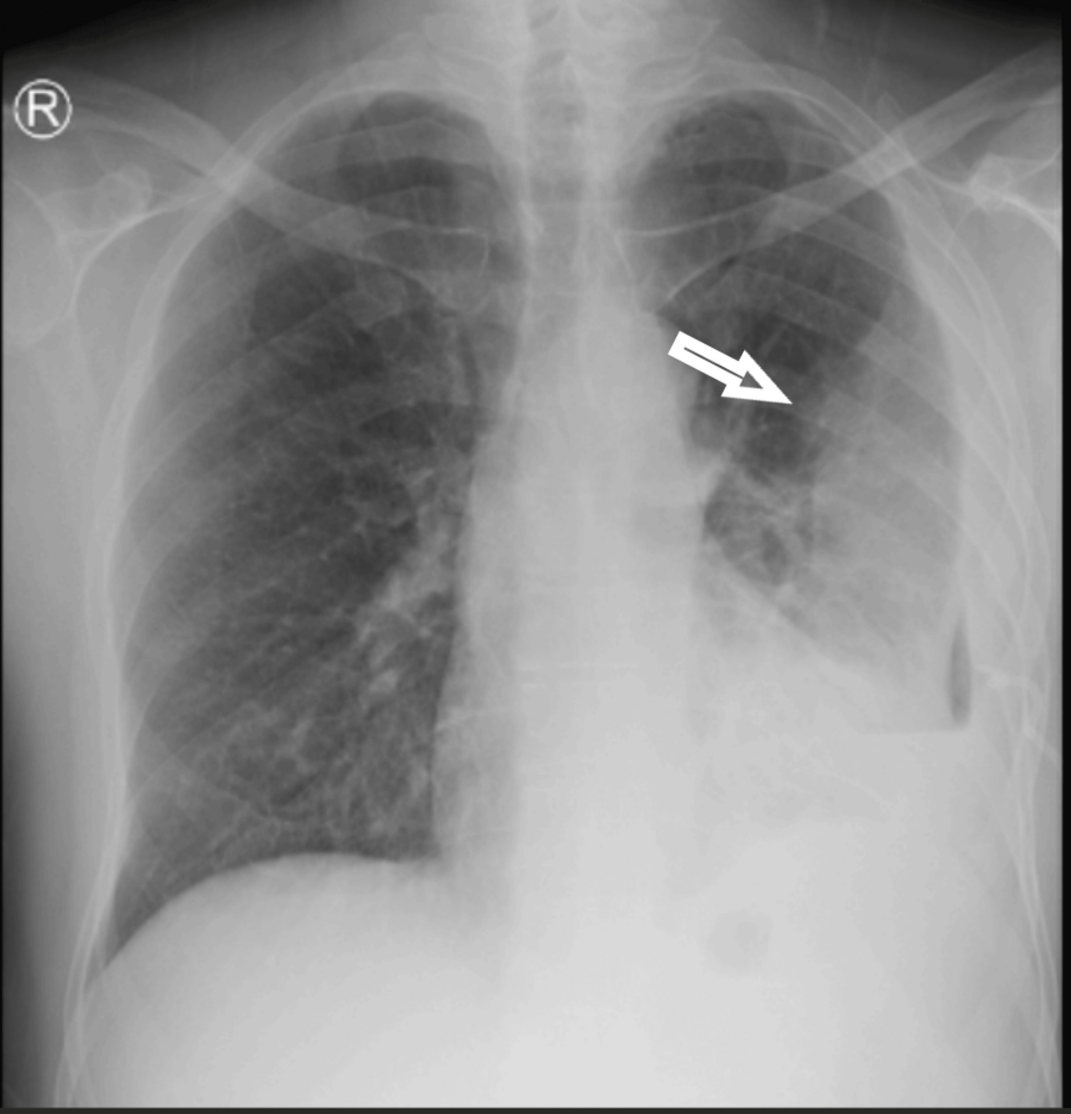
Chest X-ray. Chest X-ray demonstrates marked improvement in the left pleural effusion following pleural tapping.

Following pleural aspiration and gradual pleural fluid drainage over several days, the patient exhibited marked clinical improvement (Figure 7). The case was subsequently referred to a tertiary care facility for further staging and comprehensive management.

## Discussion

Melanoma of the breast can develop either as metastatic progression of primary cutaneous melanoma or as a primary malignant melanoma arising within the breast tissue (PMMB). While metastases to the breast from extramammary malignancies are rare, melanomas and lymphomas are the most common contributors. In male patients, breast melanoma is an exceptionally uncommon clinical entity, with most cases representing metastatic dissemination from a primary cutaneous melanoma [10].

This case underscores the need for vigilant surveillance, including the use of the ABCD criteria, in patients with a history of melanoma, particularly those in high-risk populations, such as individuals with a personal or first-degree family history of melanoma or those exhibiting the red hair phenotype. Evidence suggests that secondary prevention improves survival rates through self-examinations and clinician-led assessments using the ABCD mnemonic: asymmetry, border irregularity, color variation, diameter >6 mm, and lesion evolution, as endorsed by the Canadian and Australian cancer societies [11,12].

Breast melanoma typically presents as a palpable, non-painful mass; however, in advanced stages, clinical manifestations depend on the extent of metastatic spread. Metastatic melanoma to the pleura is rare, with malignant pleural effusions occurring in only 2% of patients with thoracic metastases [13]. When present, pleural metastases may manifest as pleural thickening or effusion, which can be unilateral or bilateral, variable in volume, and occasionally black in appearance if melanocytes are present. Malignant pleural effusions are typically exudative, with approximately 60% of cases detectable through pleural fluid cytology [14].

In our case, the primary presentation was progressive dyspnea over several weeks rather than a breast mass, an atypical presentation of breast melanoma. Additionally, pleural fluid cytology was negative for malignant cells, despite imaging and biopsy confirming metastatic disease. This discrepancy may be due to tumor cell fragility, sampling limitations, or the presence of a predominantly fibrotic or inflammatory microenvironment. Nonetheless, approximately 60% of malignant pleural effusions demonstrate cytological positivity. These findings underscore the importance of assessing suspicious breast lesions using the BI-RADS scoring system and performing tissue biopsy for accurate evaluation and staging.

Approximately 90% of melanoma cases are diagnosed as localized primary tumors without metastasis, with a 10-year survival rate of 75-95%. The primary treatment for localized melanoma is surgical resection with an adequate margin to ensure complete removal. Adjuvant therapies, including immunotherapy and targeted therapy, are commonly employed to improve survival outcomes. Systemic therapy remains the standard approach for metastatic melanoma. Historically, chemotherapy was the primary systemic treatment, with dacarbazine being the only FDA-approved drug, despite its low response rate (12.1%-17.6%). Currently, chemotherapy is reserved for patients who do not respond to immunotherapy or targeted therapy. First-line treatment now includes immune checkpoint inhibitors, irrespective of BRAF mutational status, or a combination of BRAF and MEK inhibitors for BRAF-mutant melanoma. Combining anti-PD-1 (programmed cell death protein 1) and anti-CTLA-4 (cytotoxic T-lymphocyte-associated protein 4) antibodies has demonstrated superior efficacy compared to monotherapy, with approximately half of patients with advanced melanoma achieving long-term benefits from immunotherapy. A clinical trial comparing nivolumab (anti-PD-1) and ipilimumab (anti-CTLA-4) in previously untreated, unresectable advanced melanoma reported a 6.5-year overall survival benefit [15].

In our report, the management approach included diagnostic evaluation, symptomatic relief, and bridging to systemic therapy. Upon presentation to our secondary center, the patient exhibited a unilateral loculated pleural effusion and had a history of resected cutaneous melanoma. Symptom relief was achieved through pleural tapping and gradual fluid drainage over two weeks. Imaging revealed a left breast lesion along with multiple left axillary lymph nodes, warranting a tissue biopsy, which subsequently confirmed invasive breast melanoma. After clinical stabilization, the patient was referred to a tertiary center for further staging and systemic treatment.

## Conclusions

The prognosis for metastatic melanoma remains poor, with a high fatality rate and limited treatment options. However, advances in targeted therapies and immunotherapy have significantly improved survival outcomes. Melanoma metastasizing to the breast in males is rare, and metastatic invasive melanoma involving the pleura is even more uncommon. Given the importance of early detection, regular self-examinations and clinician-led assessments are recommended for high-risk individuals with a history of resected melanoma, as they facilitate early diagnosis and improve survival rates. Clinicians should also consider metastatic melanoma in patients with a prior history of cutaneous melanoma, even in cases with atypical presentations such as isolated pleural effusion.
